# Central arterial stiffness is increased among subjects with severe and very severe COPD: report from a population-based cohort study

**DOI:** 10.3402/ecrj.v2.27023

**Published:** 2015-03-16

**Authors:** Linnea Qvist, Ulf Nilsson, Viktor Johansson, Kjell Larsson, Eva Rönmark, Jeremy Langrish, Anders Blomberg, Anne Lindberg

**Affiliations:** 1 Institute of Environmental Medicine, Karolinska Institutet, Stockholm, Sweden; 2 Department of Public Health and Clinical Medicine, Division of Medicine, Umeå University, Umeå, Sweden; 3 OLIN Unit, Department of Public Health and Clinical Medicine, Occupational and Environmental Medicine, Umeå University, Umeå, Sweden; 4 BHF Centre for Cardiovascular Science, University of Edinburgh, Edinburgh, United Kingdom

**Keywords:** COPD, epidemiology, cardiovascular disease, arterial stiffness

## Abstract

**Introduction:**

Cardiovascular disease (CVD) is common in chronic obstructive pulmonary disease (COPD) and is, as productive cough, related to poorer prognosis in COPD. Central arterial stiffness is a marker of early atherosclerosis, but the association between COPD, productive cough, and arterial stiffness as a possible indicator of CVD is unclear.

**Objectives:**

To compare both arterial stiffness among subjects with and without COPD and the impact of productive cough in a population-based cohort.

**Methods:**

A population-based cohort, including 993 COPD and 993 non-COPD subjects, has been invited to annual examination since 2005. In 2010, 947 subjects, of which 416 had COPD (according to the GOLD spirometric criteria), participated in examinations including structured interview, spirometry, and measurements of central arterial stiffness as pulse wave velocity (PWV).

**Results:**

PWV was higher in GOLD 3–4 compared to non-COPD (10.52 vs. 9.13 m/s, *p*=0.042). CVD and age ≥60 were both associated with significantly higher PWV in COPD as well as in non-COPD. In COPD, those with productive cough had higher PWV than those without, significantly so in GOLD 1 (9.59 vs. 8.92 m/s, *p*=0.024). In a multivariate model, GOLD 3–4 but not productive cough was associated with higher PWV, when adjusted for sex, age group, smoking habits, blood pressure, CVD, and pulse rate.

**Conclusions:**

GOLD 3–4, age ≥60, and CVD were associated with increased arterial stiffness, and also increased in COPD subjects with productive cough compared to those without. Of importance, GOLD 3–4 but not productive cough remained associated with increased central arterial stiffness when adjusted for confounders.

Chronic obstructive pulmonary disease (COPD) is expected to be the fourth greatest burden of disease globally by the year 2030 (1). The prevalence is estimated at about 10% (2–5) and is highly dependent on age and smoking habits; up to 50% of elderly smokers fulfill the spirometric criteria for COPD (2). In the population, a majority of those with COPD have mild to moderate disease, whereas severe to very severe disease is limited to a few percent (3). It is known that COPD is largely under-diagnosed; only approximately 30% of all subjects with COPD are identified, and the under-diagnosis is related to disease severity (3–5).

Subjects with COPD have an increased risk for Cardiovascular disease (CVD) (6), and CVDs are the most common co-morbid conditions in COPD (7, 8). Today, COPD is considered a heterogeneous syndrome with systemic effects, and the bronchitis, i.e. productive cough, in COPD is associated with increased airway inflammation, more exacerbations, and worse prognosis (9). COPD with bronchitis is probably also related to a higher prevalence of CVD than COPD without bronchitis (7). Concomitant CVD among subjects with COPD is associated with an increased mortality (10–12); however, the under-diagnosis of COPD contributes to a lack of knowledge, and probably an underestimation of the actual co-morbidity and mortality amongst individuals with COPD (12). Hence, identification of early signs of concomitant CVD in patients with COPD could contribute to improved preventive actions and better prognosis.

Central arterial stiffness has been considered a marker of early atherosclerosis (13). Increased central arterial stiffness is an independent predictor of cardiovascular mortality in healthy volunteers and patient groups alike, contributing to increased myocardial ischemia, an increased risk of stroke, and atherosclerotic plaque rupture, as a result of changes in pulse pressure (14). Small observational studies have demonstrated increased central arterial stiffness in patients with obstructive lung function (15) and that individuals with moderate to very severe COPD have increased arterial stiffness compared to controls without COPD (16). Further, increased arterial stiffness seems to be related to airway inflammation during COPD exacerbations (17). In a recently published review, measurements of arterial stiffness are suggested to be implemented in routine care for patients with COPD, whereas increased central arterial stiffness has the potential to be a useful marker of early CVD in COPD (18). However, besides a Danish population-based study (19), population level data regarding the association between arterial stiffness and COPD is limited. In the Danish study, there was a weak positive association between arterial stiffness and COPD among men under the age of 60 years when subjects with mild COPD were excluded (19).

The aim of this study was to use cross-sectional data from a population-based cohort for comparison of central arterial stiffness measurements in subjects with and without COPD and also in relation to COPD disease severity. A secondary aim was to evaluate whether productive cough was associated with increased central arterial stiffness.

## Methods

### Study population

All subjects with COPD were identified from four population-based adult cohorts from the OLIN (Obstructive Lung Disease in Northern Sweden) studies, together with age- and gender-matched subjects without obstructive lung function impairment. Since 2005, the study population (*n*=1,986) has been invited to annual examinations with a basic program including spirometry and a structured interview (20). The current study is based on data collected in 2010 when measurement of central arterial stiffness was added to the basic program. The Regional Ethics Committee at Umeå University, Sweden, approved the study.

**Fig. 1:**
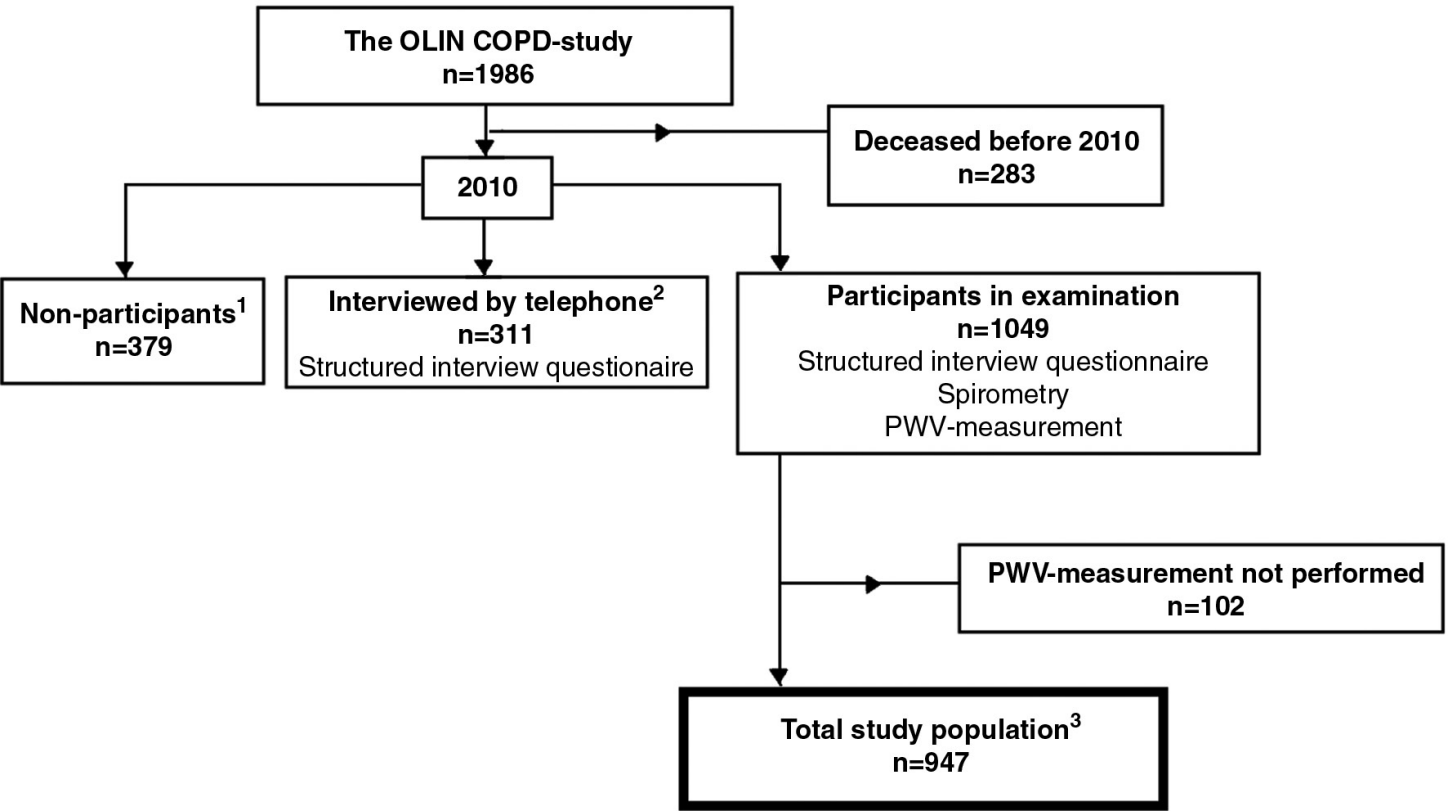
Flowchart of the recruitment of the study population. ^1^Not willing to participate. ^2^Not able to attend the examination. ^3^In 17 cases, spirometry data was missing and collected from the previous year.

As of December 31, 2009, 283 out of the original 1,986 subjects had deceased, leaving 1,703 subjects available for examination in 2010. In total, 1,324 subjects participated (78% of those alive), whereof 311 individuals who were not able to attend the examinations were interviewed by telephone. In total, 947 subjects participated in measurements of central arterial stiffness (Fig. 1).

### Structured interview

The questions regarding respiratory symptoms were developed from the British Medical Research Council questionnaire and have been validated in several studies (21, 22). In addition, self-reported data on smoking habits, respiratory diseases, and non-respiratory diseases were collected. The ‘heart disease’ variable included angina pectoris, previous coronary artery bypass surgery, previous percutaneous coronary intervention, myocardial infarction, and heart failure. CVD was defined as any heart disease, stroke, intermittent claudication, or hypertension. ‘Productive cough’ was defined as cough with phlegm on most days during a period of at least 3 months during the preceding 12 months.

### Classification of COPD

Spirometry was performed using the Vicatest 5 (Mijnhardt, The Netherlands) following the American Thoracic Society/European Respiratory Society (ATS/ERS) standards (23). Reversibility testing was performed if FEV_1_/(FVC or VC)<0.70 or FEV_1_<80% of predicted. COPD was defined by the spirometric criteria FEV_1_/best of VC or FVC<0.70, using the highest value pre- or post-bronchodilatation. Disease severity was classified according to the Global Initiative for Chronic Obstructive Lung Disease (GOLD) guidelines (24); GOLD 1–4 was based on post-bronchodilation FEV_1_ percent of predicted value. Swedish spirometric reference values were used (25).

### Central arterial stiffness

Carotid-femoral pulse wave velocity (PWV) is considered to be the ‘gold standard’ for estimating arterial stiffness (18). In this study, central arterial stiffness was measured as PWV using the non-invasive Vicorder^®^ system (Skidmore Medical Ltd., UK), which detects the pressure wave simultaneously at the carotid and femoral arteries using inflatable cuffs. The pulse transit time between these sites is calculated by identifying the arrival of the foot of the pulse wave using a proprietary intersecting tangents algorithm and comparing it to the R-wave on the electrocardiogram. The anatomical distance between the sites divided by pulse transit time is then used to generate the PWV. PWV was measured after resting for 20 min and recorded three times within 5–10 min. At least two recordings within a 0.5 m/s range were necessary for the recordings to be considered reproducible; otherwise recordings were repeated up to a total of five recordings. The median PWV was estimated for each individual.

Blood pressure was measured manually in the left arm with the subjects in supine position, after 20 min of rest, prior to measurement of PWV.

### Statistical analyses

Statistical analyses were performed using Statistical Package for the Social Sciences (SPSS, Software Version 18). A *p*-value of <0.05 was considered statistically significant. Mean PWV values were based on median values for each subject. The median of individual levels was used to eliminate the influence of extreme values. PWV data were explored by evaluating histograms and were considered normally distributed. Descriptive statistics was used and comparisons between groups were performed by chi-2-test, independent sample *t*-test, and ANOVA for comparing means; Bonferroni correction was used for multiple testing. Median PWV was analyzed in relation to COPD disease severity according to GOLD in a linear regression model, including the independent variables gender, age-group (<60 or ≥60 years), and smoking habits. In Models A–C, blood pressure (systolic, diastolic, and both, respectively) was added, and in Model D systolic blood pressure and the CVD variable were added. Productive cough and pulse rate were also added to each of the models.

## Results

### Study population

In total, 416 subjects with COPD and 531 without COPD were included. There was no significant difference in the prevalence of co-morbidities when comparing subjects with and without COPD. All respiratory symptoms, including productive cough, were more common in COPD subjects compared to non-COPD subjects (Table 1).

**Table 1:** Basic characteristics of study group, showing COPD and non-COPD, number of subjects=*n* (percent)

	Non-COPD	COPD	*p*^a^

	*n*=531	*n*=416	
Gender			0.710
Male	300 (56.5)	230 (55.3)	
Female	231 (43.5)	186 (44.7)	
Age			
Mean (±SD)	68.1 (10.1)	68.8 (9.4)	0.249
<60	96 (18.1)	75 (18.0)	
≥60	435 (81.9)	341 (82.0)	0.984
BMI^b^			
Mean (±SD)	27.6 (4.1)	26.8 (4.2)	0.005
<18.5	1 (0.2)	3 (0.7)	0.002
18.5–<24.9	128 (24.1)	143 (34.4)	
25–<29.9	294 (55.4)	189 (45.4)	
≥30	108 (20.3)	81 (19.5)	
Smoking habits			<0.001
Non-smoker	258 (48.8)	112 (27.1)	
Ex-smoker	225 (42.5)	209 (50.5)	
Current smoker	46 (8.7)	93 (22.5)	
COPD disease severity			
GOLD 1		266 (63.9)	
GOLD 2		137 (32.9)	
GOLD 3–4		13 (3.1)	
Respiratory symptoms			
Productive cough	82 (15.5)	98 (23.7)	0.002
Attacks of breathlessness	52 (9.9)	88 (21.2)	<0.001
Wheezing/whistling during last 12 months	28 (5.3)	46 (11.1)	0.001
Any respiratory symptom	181 (34.1)	242 (58.2)	<0.001
Co-morbidities			
Heart disease	90 (16.9)	68 (16.3)	0.805
Hypertension	255 (48.0)	194 (46.6)	0.671
Claudication	7 (1.3)	7 (1.7)	0.643
Stroke	22 (4.2)	26 (6.3)	0.139
CVD^c^	287 (54.0)	225 (54.1)	0.991

a Comparing COPD and non-COPD

b BMI=body mass index

c CVD=cardiovascular disease. Age and BMI are presented as mean (±SD).

In the non-COPD group, those with productive cough had significantly higher prevalence of CVD compared to those without productive cough (50.4% vs. 75.6%, *p*<0.001), whereas there was no such difference among subjects with COPD (54.3% vs. 54.1%, *p*=0.972). Among all subjects with productive cough, non-COPD subjects had a significantly higher prevalence of CVD compared to COPD subjects (75.6% vs. 54.1%, *p*=0.003).

Baseline data from 2002 to 2004 was compared between those who died before December 31, 2009 (*n*=283), and subjects participating in the 2010 examination. The deceased subjects were older, more often men, and had a higher prevalence of COPD, productive cough, and heart disease (Table 2).

**Table 2:** Baseline characteristics of subjects deceased by December 31, 2009, compared to those participating in examination with arterial stiffness

	Participants	Deceased	*p* a
	
	*n*=947	*n*=283	
Age (years)	61	74	<0.001
Gender (% male)	56.0	63.3	0.030
COPD (%)	47.3	58.3	0.001
Productive cough (%)	30.3	47.3	<0.001
Heart disease (%)	20.8	44.5	<0.001

a Comparing baseline data from 2002 to 2004 for those who died by December 31, 2009, and for those subjects attending examinations including arterial stiffness.

### Central arterial stiffness and COPD

PWV was increased in subjects with GOLD 3–4 as compared to subjects with GOLD 1, GOLD 2, and significantly so when compared to non-COPD subjects (10.52 vs. 9.13, *p*=0.042) (Table 3). Univariate analyses identified increased PWV to be significantly associated with factors such as age ≥60 and CVD, whereas there were no statistical significant sex differences. In both COPD, all GOLD stages, and non-COPD, smokers had the lowest mean PWV, significantly lower than non-smokers among non-COPD-subjects (Table 3). Among COPD subjects, PWV was higher in those with productive cough compared to those without productive cough (9.44 vs. 9.00, *p*=0.052), and the difference reached statistical significance in GOLD 1 (9.59 vs. 8.92, *p*=0.024) (Table 3).

**Table 3:** Mean PWV (m/s,±SD) in non-COPD, COPD, and COPD by disease severity presented by gender, age group, smoking habits, with and without productive cough (prod cough), and with and without CVD

	Non-COPD	COPD	*p* b	GOLD 1	*p* c	GOLD 2	*p* c	GOLD 3–4	*p* c
			
	*n*=531	*n*=416		*n*=266		*n*=137		*n*=13	
PWV	9.13 (2.06)	9.12 (1.95)	0.916	9.07 (1.96)	0.976	9.06 (1.79)	0.980	10.52 (2.95)	0.042
Gender									
Male	9.25 (1.87)	9.17 (1.97)	0.606	9.12 (2.03)	0.860	9.10 (1.61)	0.881	11.25 (3.64)	0.033
Female	8.97 (2.29)	9.05 (1.94)	0.691	9.02 (1.89)	0.995	9.01 (2.02)	0.998	9.89 (2.31)	0.593
*p* d	0.110	0.540		0.675		0.787		0.429	
Age groups									
<60	7.85 (1.63)	7.80 (1.14)	0.838	7.75 (1.25)	0.910	7.87 (0.96)	0.997	8.30*	–
≥60	9.41 (2.05)	9.40 (1.98)	0.956	9.34 (1.97)	0.966	9.38 (1.82)	0.999	10.70 (3.00)	0.083
*p* e	<0.001	<0.001		<0.001		<0.001		–	
Smoking habits									
Non-smoker	9.33 (2.23)	9.04 (2.12)	0.249	8.98 (2.07)	0.491	9.06 (2.03)	0.917	11.50 (4.53)	0.415
Ex-smoker	9.07 (1.84)	9.37 (1.88)	0.090	9.30 (1.80)	0.554	9.36 (1.79)	0.542	11.63 (4.33)	0.019
*p*^f^	0.285	0.256		0.389		0.657		0.998	
Current smoker	8.29 (1.96)	8.66 (1.86)	0.280	8.63 (2.11)	0.748	8.53 (1.54)	0.901	9.60 (1.57)	0.232
*p* f	0.003	0.274		0.517		0.383		0.622	
Respiratory symptoms									
Without prod cough	9.18 (2.13)	9.00 (1.91)	0.241	8.92 (1.97)	0.341	9.08 (1.75)	0.965	10.04 (2.16)	0.501
With prod cough	8.87 (1.66)	9.44 (2.05)	0.044	9.59 (1.84)	0.077	9.02 (1.90)	0.956	11.59 (4.48)	0.014
*p* g	0.214	0.052		0.024		0.869		0.405	
Co-morbidities									
Without CVD^a^	8.68 (1.94)	8.61 (1.73)	0.714	8.52 (1.77)	0.814	8.77 (1.63)	0.981	9.26 (1.93)	0.893
With CVD	9.51 (2.09)	9.54 (2.03)	0.874	9.59 (1.99)	0.979	9.29 (1.88)	0.764	11.07 (3.24)	0.075
*p* h	<0.001	<0.001		<0.001		0.092		0.328	

a CVD=cardiovascular disease

b comparing between the groups: non-COPD and COPD

c non-COPD and each GOLD grade (using Bonferroni correction for multiple testing)

d comparing gender

e comparing age groups

f comparing non-smoker with ex-smoker and non-smoker with current smoker

g comparing with and without productive cough

h comparing with and without cardiovascular disease

* only one subject.

### Multivariate analysis

The distribution of PWV was reasonably normally distributed, and the association between PWV and GOLD stages was analyzed by linear regression analyses (Table 4), in a model including blood pressure and CVD in different combinations (Models A–D). GOLD 3–4 remained consistently significantly associated with higher PWV throughout all models. Age ≥60 years was associated with higher PWV, while current smoking was associated with lower PWV in all models. Both systolic and diastolic blood pressure were significantly associated with increasing PWV when separately added (Models A and B), but only systolic pressure remained significantly associated with PWV when simultaneously added (Model C). CVD and systolic blood pressure were both significantly associated with higher PWV when included simultaneously (Model D) (Table 4). Productive cough was not associated with PWV when included in the models, and the presence of productive cough did not have any impact on the observed significant associations. There was no significant interaction between smoking status and age group. Replacing the variable smoking status with pack-years in the models did not significantly alter the findings. Adjusting for pulse rate also did not significantly alter the findings in Models A-D, and pulse rate was significantly associated with higher PWV in all models. All correlations between the independent variables were <0.5 and the F-test remained significant for all models. No obvious signs of multicollinearity were detected. There were some tendencies to heteroscedasticity in Models B-D after the inclusion of pulse rate into the models.

**Table 4:** Multivariate models analyzing the association between PWV and COPD GOLD grades, adjusting for sex, age group, and smoking habits, B-coefficient and 95% confidence interval (CI)

	Model A		Model B		Model C		Model D	
			
	B-coef	95% CI	B-coef	95% CI	B-coef	95% CI	B-coef	95% CI
Sex^a^	0.147	−0.095–0.388	0.207	−0.040–0.453	0.138	−0.104–0.380	0.160	−0.080–0.400
Age ≥60 years^b^	1.379***	1.067–1.690	1.579***	1.261–1.898	1.323***	1.003–1.642	1.243***	0.926–1.559
Ex smoker^c^	−0.044	−0.380–0.221	−0.080	−0.351–0.191	−0.046	−0.310–0.219	−0.072	−0.355–0.191
Smoker^c^	−0.584***	−0.963–−0.206	−0.675**	−1.061–−0.288	−0.562**	−0.941–−0.182	−0.568**	−0.944–−0.193
GOLD 1^d^	0.091	−0.187–0.369	0.024	−0.259–0.308	0.098	−0.180–0.375	0.098	−0.177–0.374
GOLD 2^d^	0.065	−0.296–0.425	0.088	−0.281–0.457	0.069	−0.291–0.429	0.048	−0.310–0.406
GOLD 3–4^d^	1.591**	0.559–2.623	1.433**	0.377–2.490	1.648**	0.614–2.682	1.518**	0.494–2.543
Syst BP^e^	0.027***	0.021–0.034	not incl		0.032***	0.023–0.042	0.026***	0.019–0.032
Diast BP^e^	not incl		0.028***	0.016–0.040	−0.013	−0.029–0.004	not incl	
CVD	not incl		not incl		not incl		0.490***	0.245–0.735

a Reference men

b reference age <60 years

c reference non-smoker

d reference non-COPD

e continuous variable

** *p*<0.010

*** *p*<0.001.

To Models A–C, blood pressure (BP), systolic, diastolic, and both, respectively, were added, and to Model D systolic blood pressure and reported CVD were added.

## Discussion

In this population-based study, subjects with severe and very severe COPD, GOLD 3–4, had significantly increased central arterial stiffness when compared with non-COPD subjects. In all subjects, independent of COPD, both higher age and reported CVD were significantly associated with increased PWV. Subjects with COPD and productive cough had higher PWV than COPD subjects without productive cough. However, GOLD 3–4 but no productive cough remained significantly associated with increased central arterial stiffness, when adjusted for confounders such as sex, age, smoking habits, measured blood pressure, pulse and reported CVD.

Our data support the findings of previously reported observational data generated from selected COPD populations. In a study of around 100 subjects with predominantly severe and very severe COPD and a similar size group of healthy controls, central arterial stiffness increased with age and was increased in COPD subjects compared to non-COPD subjects (16). In another study including nearly twice as many subjects with COPD (mean FEV_1_ 50% of predicted), arterial stiffness increased by age and was higher in men compared to women (26). Increased arterial stiffness was also related to atherosclerotic manifestation in a study of 119 subjects with moderate to severe COPD (27). Even though these studies used the ‘gold standard’, PWV, to assess arterial stiffness, direct comparison with our study is difficult as the studies used a different method and device, the SphygmoCor^®^ (AtCor Medical, Australia), to measure arterial stiffness. Further, no control groups without COPD were included in the last two studies.

To the best of our knowledge, only one large population-based study evaluating the relationship between arterial stiffness and COPD has so far been published (19). In this Danish study, a weak association between arterial stiffness and COPD was found, though only in men below the age of 60 and when mild COPD (GOLD 1) was excluded. In contrast to the Danish study (19), we found that arterial stiffness was significantly increased among subjects with GOLD 3–4 compared to non-COPD subjects, also when adjusted for confounders. However, in the Danish study arterial stiffness was assessed by the aortic augmentation index (AIx), a more indirect global measure of arterial stiffness than PWV. Although AIx is dependent upon PWV, it is not possible to make a direct comparison between the two different methods of assessing arterial stiffness.

A consistent finding in our study was a lower arterial stiffness in smokers compared to in non-smokers and ex-smokers. In addition, the multivariate model showed a stable association between current smoking and decreased arterial stiffness, and there was no interaction between smoking status and age. Previous reports are contradictory: two studies have reported no association between smoking habits and arterial stiffness (15, 16), whereas current smoking was associated with an increase in arterial stiffness in the previously mentioned Danish population-based study (19). Our finding was unexpected, but could be related to a ‘healthy smoker effect’; i.e. smokers with a disease burden such as CVD may have been more prone to stop smoking, whereas those without these conditions continued smoking.

Productive cough in COPD is associated not only with exacerbations (28), but also with CVD and increased mortality (7, 11, 12). It has also been suggested that exacerbations help to identify and diagnose previously unknown COPD, contributing to initiation of proper medication (29). Overspill of airway inflammation to systemic inflammation is suggested as a possible link to co-morbidities in COPD (30), and increased central arterial stiffness seems to be related to inflammation in COPD exacerbations (17). Until now, arterial stiffness has not been evaluated in relation to clinical signs of airway inflammation such as productive cough. We found increased central arterial stiffness among subjects with COPD and productive cough, significantly so in GOLD 1, when compared to those COPD subjects without productive cough, even though the prevalence of CVD was similar in both groups. Consequently, increased central arterial stiffness in COPD subjects with productive cough cannot be explained by the presence of known CVD, but may reflect early signs of as-yet undiagnosed disease. However, when included in the multivariate models, productive cough was no longer a significant risk factor for increased PWV. In a recently published review it was suggested that arterial stiffness should be measured during routine care of COPD patients, as an indicator of early CVD (18). With further population-based studies including a large number of GOLD 1 and GOLD 2 subjects, we may find subgroups with higher risk of CVD. Subjects with increased inflammatory burden such as bronchitis or exacerbations may be of special interest.

The strength of this population-based study is the large number of subjects with COPD identified by spirometry. The high participation rate, the standardized methods, and the distribution of COPD disease severity, corresponding well to what is known from population-based studies (3), contribute to an assumption of high internal as well as external validity. Although a population-based sample will include only a small proportion of GOLD 3–4, the differences in PWV were large enough to demonstrate that severe and very severe COPD remained a significant risk factor for increased PWV also in the multivariate models.

PWV is considered the ‘gold standard’ of measuring arterial stiffness (18), but there are different devices to measure PWV. In this study we used the Vicorder^®^ device, but another commonly used device is the SphygmoCor^®^ (16, 26, 27). The two devices have been evaluated in comparative studies. The reproducibility of the Vicorder^®^ was reported inferior to the SphygmoCor^®^ in a small study including 38 subjects (31), but in a larger study with three times as many subjects the measurements were considered highly repeatable and correlated well between the Vicorder^®^ and the SphygmoCor^®^ (32). An advantage of the Vicorder^®^ is that it is less operator-dependent than the SphygmoCor^®^ and may thus be more suitable for use in epidemiological fieldwork (31, 32).

CVD is related to atherosclerosis, therefore we did not separate out heart disease, stroke, claudication, and hypertension when evaluating central arterial stiffness. However, one limitation is that data on CVD was self-reported and not verified by medical records. Analysis of mortality data, from the initial recruitment of the study population through December 2009, strengthens the assumption of a healthy survival effect: the deceased subjects, besides being older as expected, also had a higher frequency of COPD, productive cough, and heart disease. The results may, taking the strengths and weaknesses into account, be considered generalizable to subjects with COPD in the general population and, because of the healthy survivor effect, the observed relationships with central arterial stiffness may actually be underestimated.

In conclusion, in this large population-based cohort, central arterial stiffness was increased among subjects with severe and very severe COPD when compared to non-COPD subjects. Further, central arterial stiffness was increased among COPD subjects with productive cough compared to those without, which may indicate that early atherosclerosis is more common in the bronchitis phenotype of COPD. However, severe and very severe COPD, but not productive cough, remained a risk factor for increased central arterial stiffness when adjusted for possible confounders. Further studies are needed to evaluate central arterial stiffness as a possible marker of early and undiagnosed CVD in COPD, and a longitudinal follow-up is important for the understanding of central arterial stiffness as prognostic marker for early CVD in COPD.
